# Metabolomics analysis of the effect of GnRH on the pregnancy rate of ewes with estrus synchronization scheme based on progesterone

**DOI:** 10.3389/fvets.2024.1442931

**Published:** 2024-07-11

**Authors:** Jing Zhang, Shuyuan Sun, Xinyu Bai, Nana Yang, Yiyong Liu, Xinglong Wu, Xiangyun Li

**Affiliations:** ^1^College of Animal Science and Technology, Hebei Technology Innovation Center of Cattle and Sheep Embryos, Hebei Agricultural University, Baoding, Hebei, China; ^2^College of Animal Science, Tarim University, Alear, Xinjiang, China; ^3^Institute of Xinjiang Yili Animal Science, Yining, Xinjiang, China

**Keywords:** gonadotropin-releasing hormone, pregnancy rate, metabolomics, hydroxyproline, prostaglandin D2, corticosterone, ewes

## Abstract

**Introduction:**

Gonadotropin-releasing hormone (GnRH) is widely used in the timed artificial insemination protocol for sheep. However, there remains a debate regarding its impact on pregnancy rates during artificial insemination. This study aims to evaluate the effect of GnRH on the pregnancy rates in Huyang ewes, analyze the pre-implantation metabolite changes caused by GnRH using metabolomics, and elucidate the mechanism effect on pregnancy rates.

**Methods:**

All ewes were administered a vaginal progesterone sponge containing 45 mg of flurogestone acetate for 12 days and received 330 units of equine chorionic gonadotropin (eCG) intramuscularly after sponge removal. The experimental group (*n* = 69) received an intramuscular treatment of 17 μg GnRH agonist triptorelin 48 h after sponge removal on Day 0, while the control group (*n* = 41) received 1 mL of sterile saline solution. All ewes underwent a single vaginal insemination 58 h after the withdrawal of the progesterone sponge. The difference in pregnancy rates between the two groups was calculated. Metabolomic analysis was performed on plasma samples collected on Day 7 after the treatment of GnRH agonist.

**Results:**

Gonadotropin-releasing hormone (GnRH) treatment significantly reduced the pregnancy rate in the experimental group compared with the control group (72.2 vs. 82.9%, *p* < 0.05). Metabolomic analysis indicated that GnRH treatment affected metabolites involved in collagen synthesis and prostaglandin synthesis in the endometrial tissue, which includes a marked decrease in hydroxyproline amino acid content and a significant increase in corticosterone and prostaglandin D2 lipids and unsaturated fatty acids.

**Conclusion:**

In summary, the injection of GnRH agonist Triptorelin 48 h after progesterone sponges removal reduces the pregnancy rate of Huyang ewe following artificial insemination. It also affects the metabolite levels related to endometrial collagen and prostaglandin synthesis, harming embryo implantation.

## 1 Introduction

Promoting estrus synchronization and artificial insemination technology in the sheep industry can increase lamb production, improve reproductive efficiency, and enhance flock management and genetic improvement (1). Synchronized breeding results in synchronized lambing, simplifying centralized management and lamb sales (2). In production practice, a commonly used estrus synchronization program for ewes undergoing artificial insemination or serving as recipients in the Multiple Ovulation and Embryo Transfer (MOET) process involves the use of vaginal inserts or sponges containing fluorogestone acetate or medroxyprogesterone acetate and eCG (3, 4). Numerous studies have demonstrated the effectiveness of using gonadotropin-releasing hormone (GnRH) to synchronize and induce ovulation in farm animal reproduction (5–7). It has been demonstrated by Menchaca et al. (2) that administering a single dose of GnRH 24–36 h after removing the progesterone-releasing intravaginal device results in a luteinizing hormone peak approximately 40 h later. Additionally, 90% of ewes ovulate within 72 h after removing the device.

Although GnRH has been widely used in Fixed Time Artificial Insemination (FTAI) projects for sheep (8), resulting in a certain degree of economic benefit improvement in animal husbandry production (9), its effect on pregnancy rates is still debated (2). Some studies indicated that administering a single dose of GnRH after pessary removal increases pregnancy rates (10, 11), contrasting with findings of no effect (6, 12, 13) or a decrease in pregnancy rates in other studies (14). Similarly, in cows undergoing estrus synchronization, administering GnRH during insemination leads to the ovulation of physiologically immature follicles, impacting the pregnancy rate of cows and the fetal survival in later gestation (15). Pre-ovulatory follicle exposure to the gonadotropin surge triggered by exogenous GnRH may reduce oocyte quality, decreasing the pregnancy rate (16). Moreover, it has been inferred that GnRH might hinder embryo implantation, consequently affecting pregnancy rates in FTAI protocols (17).

Metabolomics is an important technology for studying the metabolic networks of biological systems. It involves quantitatively analyzing all metabolites in organisms and exploring the relative relationships between metabolites and physiopathological changes (18). This technique is extensively applied in plant research (19), drug development (20), and disease studies (21). Various studies have utilized metabolomics to detect sheep meat quality (22), feed and nutrition (23), and diseases (24). Relevant studies have also used metabolomics to analyze the composition of ewe cervical mucus and the negative impact of inflammatory responses on the transportation of semen in the cervix (25). However, research on the implantation environment during the peri-implantation period remains limited.

In the study, Huyang ewes were treated with estrus synchronization, GnRH agonist was injected 48 h after the withdrawal of progesterone sponges, and the difference in pregnancy rate was compared 40 days after insemination. Blood samples were collected on Day 7 post-GnRH treatment for metabolomics analysis to identify metabolic pathways and metabolites impacting pregnancy rates in Huyang ewes caused by GnRH. The objective of the present study was to evaluate the effect of GnRH treatment on pregnancy rates and pre-implantation metabolites in Huyang ewes.

## 2 Materials and methods

### 2.1 Animals and location

The study was conducted in January 2023 in Hulunbeir, Inner Mongolia. It is located at longitude 120°28′E and latitude 47°5′N. This region experiences a temperate continental climate and receives annual precipitation ranging from 300 to 500 mm. Winters are chilly and arid, while summers are hot and rainy, resulting in significant fluctuations in both annual and daily temperatures. The experiment selected 23 healthy and multiparous Huyang ewes (aged 1.5–4 years, weighing 40–45 kg, BSC ≥ 3, ranging from emaciation to obesity). All the ewes participating in the experiment possess healthy reproductive tracts and lactating abilities, with an average weaning period exceeding 3 months, thus qualifying them for estrus synchronization treatment. During the experimental period, the ewes were kept away from the rams to prevent voluntary mating. The ewes were kept indoors at night and allowed to graze at natural pastures on the farm throughout the day. When ewes were kept indoors, all ewes received TMR, including alfalfa hay, barley, and mixed concentrate according to NRC recommendation, and had free access to water and mineral block. This study was approved by the Animal Care and Use Committee of the Hebei Agricultural University, Baoding, China (Number: 2021011). All efforts were made to minimize animal suffering. The authors declare that all procedures in the experiment were conducted in ways consistent with the precepts of animal welfare, with personnel involved in the caring and handling of animals being licensed veterinarians.

### 2.2 Estrus synchronization and hormonal treatment

All experimental ewes comprised indigenous, multiparous Huyang ewes, which were synchronized using a progestogen-eCG combination protocol. Each ewe received a polyurethane intravaginal sponge impregnated with 45 mg flurogestone acetate (Muqimuye Sci-Tech Co., Ltd., Shanghai, China) for 12 days, followed by treatment with 330 IU of eCG i.m. (Sansheng Biological Technology Co., Ltd., Ningbo, China) at sponge removal. The experimental group (B, *n* = 69) was subjected to 48 h (Day 0) after sponge removal to intramuscular administration of 17 μg of the GnRH agonist triptorelin (Sansheng Biological Technology Co., Ltd., Ningbo, China), and the control group (Y, *n* = 41) with 1 mL of sterile physiological saline solution. All ewes underwent a single vaginal insemination 58 h after the withdrawal of progesterone sponges.

### 2.3 Fixed-time artificial insemination

Fresh sperm was collected from six healthy Huyang rams, and sperm motility and density were assessed using a hemocytometer and an optical microscope. The sperm (progressive motility more significant than 50% and density greater than 1 × 10^9^ sperm/mL) was diluted using 5–7 times the volume of skim milk (Yili skimmed milk, Inner Mongolia Yili Industrial Group Limited by Share Ltd), ensuring that the motility of sperm after dilution remained above 0.5, and the density was adjusted to 4 × 10^8^ sperm/mL. The diluted sperm was then stored in a water bath kettle at 30°C. The experimental sheep were restrained upside down, and their vaginal mucus was cleaned with warm normal saline to ensure the hygiene of the vaginal environment, thereby reducing the impact on sperm viability. A vaginal speculum was inserted to open the vagina, and the cervical os was observed and located. A sheep-specific inseminator (produced by Baoding Zhengmu Biotechnology Co., Ltd.) was used to aspirate 0.3 mL of semen and inject it into the cervical os.

### 2.4 Blood sample collection

Ewes were randomly selected from the experimental group (*n* = 13) and the control group (*n* = 10). In the experimental group (*n* = 13) and the control group (*n* = 10), 5 mL of jugular venous blood was collected using vacuum blood collection tubes containing heparin sodium on Day 7 after the treatment of GnRH agonist. Afterward, the blood samples underwent centrifugation at 3,000 × *g* for 15 min at 4°C. The resulting supernatant plasma was carefully separated and transferred into enzyme-free 1.5 mL EP tubes using a precision pipette. The plasma samples were immediately stored at −80°C to preserve their integrity for subsequent metabolomics analysis.

### 2.5 Serum liquid chromatography–tandem mass spectrometry detection

Mix 100 μL of the sample with 400 μL of pre-cooled methanol, ensuring thorough mixing by vortexing. Incubate the mixture on ice for 5 min, centrifuge it at 15,000 × *g*, and 4°C for 20 min. Dilute a portion of the resulting supernatant with LC–MS grade water to achieve a final methanol concentration of 53%. Subsequently, transfer the diluted sample to a new centrifuge tube using a 0.22 μm filter and centrifuge it again at 15,000 × *g* and 4°C for 20 min. Finally, the filtered sample is injected into the LC–MS/MS system for analysis.

The LC–MS/MS analysis was conducted using the Vanquish UHPLC system (ThermoFisher, Germany) coupled with an Orbitrap Q ExactiveTMHF-X mass spectrometer (ThermoFisher, Germany). Samples were injected into a Hypersil Gold chromatographic column (100 mm × 2.1 mm, 1.9 μm) at a flow rate of 0.2 mL/min using a 17-min linear gradient. For the positive polarity mode, the mobile phases consisted of eluent A (0.1% Formic Acid in water) and eluent B (Methanol). For the negative polarity mode, the mobile phases were eluent A (5 mM ammonium acetate, pH 9.0) and eluent B (methanol). The solvent gradient was set as follows: 2%B, 1.5 min; 2–85%B, 3 min;85–100%B, 10 min; 100–2%B, 10.1 min; 2%B, 12 min. The Q ExactiveTMHF-X mass spectrometer was operated in both positive and negative modes with a spray voltage of 3.5 kV, a capillary temperature of 320°C, a sheath gas flow rate of 35 psi, and an auxiliary gas flow rate of 10 L/min, S-lens RF level of 60, Aux gas heater temperature of 350°C.

### 2.6 Pregnancy test

Forty days after insemination, a fixed technician used a B-ultrasound tester (DP-30Vet, Shenzhen Mindray Biomedical Electronics Co., Ltd.) to perform a pregnancy test on the inseminated ewes through the abdomen. The B-ultrasound screen showed a clear gestational sac, which was identified as pregnancy. The pregnancy rates were calculated as the percentage of pregnant ewes among the inseminated ewes.

### 2.7 Data analysis

Statistical analysis was carried out using GraphPad Prism 8.0 software. The pregnancy rates in the experimental and control groups were analyzed using a Chi-square Test. It was accepted as significant if the calculated *p* value <0.05. Use the Human Metabolome Database (HMDB, http://www.hmdb.ca/), Kyoto Encyclopedia of Genes and Genomes (KEGG, http://www.genome.jp/kegg/), and Lipidmaps (Lipid Metabolism Pathway Research Program, https://lipidmaps.org/) databases to annotate metabolites. Utilize metaX for Partial Least Squares Discriminant Analysis (PLS-DA) and employ univariate analysis (T-test) to calculate statistical significance (*p* value). PLS-DA was performed to compute Variable Importance in Projection (VIP) scores. Metabolites that fulfill the criteria of Variable Importance in Projection (VIP) > 1, *p* value <0.05, and either Fold Change (FC) > 1.2 or FC < 0.833 are deemed as differential metabolites. The functions of these metabolites and metabolic pathways were studied using the KEGG database. The metabolic pathways enrichment of differential metabolites was performed, and metabolic pathways were considered enrichment. When the *p* value of metabolic pathway <0.05, metabolic pathways were considered statistically significant enrichment.

## 3 Results

### 3.1 Effect of GnRH on pregnancy rates at 40 days after insemination

Pregnancy was detected by ultrasound 40 days after insemination, the result as shown in Table 1. The pregnancy rate of the experimental group was significantly lower than that of the control group (72.2 vs. 82.9%, *p* < 0.05). Results showed that GnRH treatment during artificial insemination decreased the pregnancy rates in Huyang ewes.

**Table 1:** The effect of GnRH treatment on pregnancy rate in Huyang ewes.

Group	No. Inseminated ewes	No. Pregnant ewes	Pregnancy rate (%)
Experimental group	69	50	72.5^*^
Control group	41	34	82.9

The pregnancy rates were analyzed using a Chi-square Test. ^*^p < 0.05.

### 3.2 Classification of metabolites in ewes

**Figure 1:**
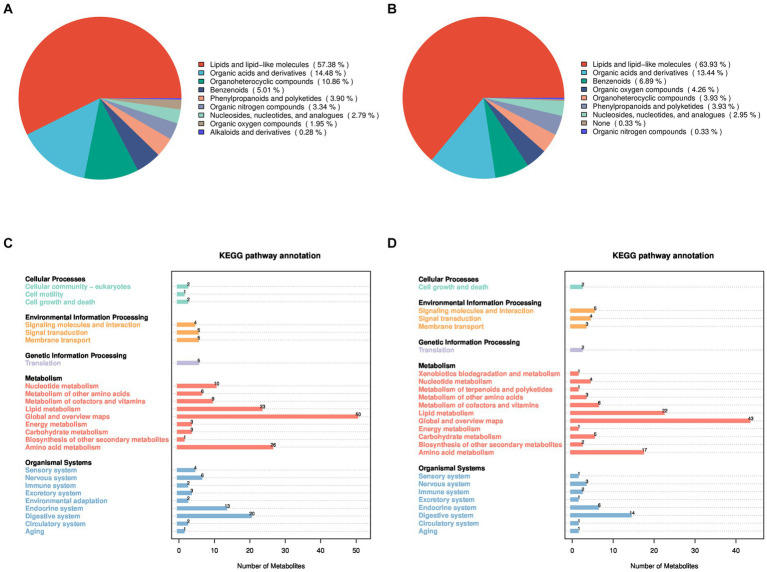
Identification and classification of all metabolites in Huyang ewe plasma samples. Chemical classification of all metabolites in the positive ion mode **(A)** and the negative ion mode **(B)**. On the left is a pie chart, and on the right is the proportion of metabolites. KEGG functional annotation of all metabolites in the positive ion mode **(C)** and the negative ion mode **(D)**, on the left is the metabolite-enriched pathway, and on the right is the number of metabolites in the pathway.

To investigate the factors contributing to the reduced pregnancy rates following GnRH treatment in Huyang ewes, plasma samples from both experimental and control groups were collected 7 days after artificial insemination for metabolomics. All 23 samples identified 464 metabolites in positive ion mode (POS) and 376 in negative ion mode (NEG). Positive ion mode analysis revealed a range of metabolites such as lipids and lipid-like molecules (57.38%), organic acids and derivatives (14.48%), organoheterocyclic compounds (10.86%), benzenoids (5.01%), phenylpropanoids and polyketides (3.90%), organic nitrogen compounds (3.34%), nucleosides, nucleotides, and analogs (2.79%), organic oxygen compounds (1.95%), and alkaloids and derivatives (0.28%) (Figure 1A). Negative ion mode analysis indicated the existence of lipids and lipid-like molecules (63.93%), organic acids and derivatives (13.44%), benzenoids (6.89%), organic oxygen compounds (4.26%), organoheterocyclic compounds (3.93%), phenylpropanoids and polyketides (3.93%), nucleosides, nucleotides, and analogs (2.95%), none (0.33%), and organic nitrogen compounds (0.33%) (Figure 1B).

In addition, the KEGG database was utilized for functional annotation of the identified metabolites to comprehend their functional traits and classifications (Figures 1C,D). In the positive and negative modes, the identified metabolites participate in pathway processes related to cellular processes, environmental information processing, genetic information processing, metabolism, and organic systems. Specifically, under the positive ion mode, metabolites primarily participate in biological processes such as cell growth and death, membrane transport, lipid metabolism, energy metabolism, and amino acid metabolism. In the negative ion mode, metabolites mainly involve biological processes, including cell growth and death, signaling molecules and interaction, lipid metabolism, amino acid metabolism, and the immune system.

### 3.3 Screening and analysis of differential metabolites

**Figure 2:**
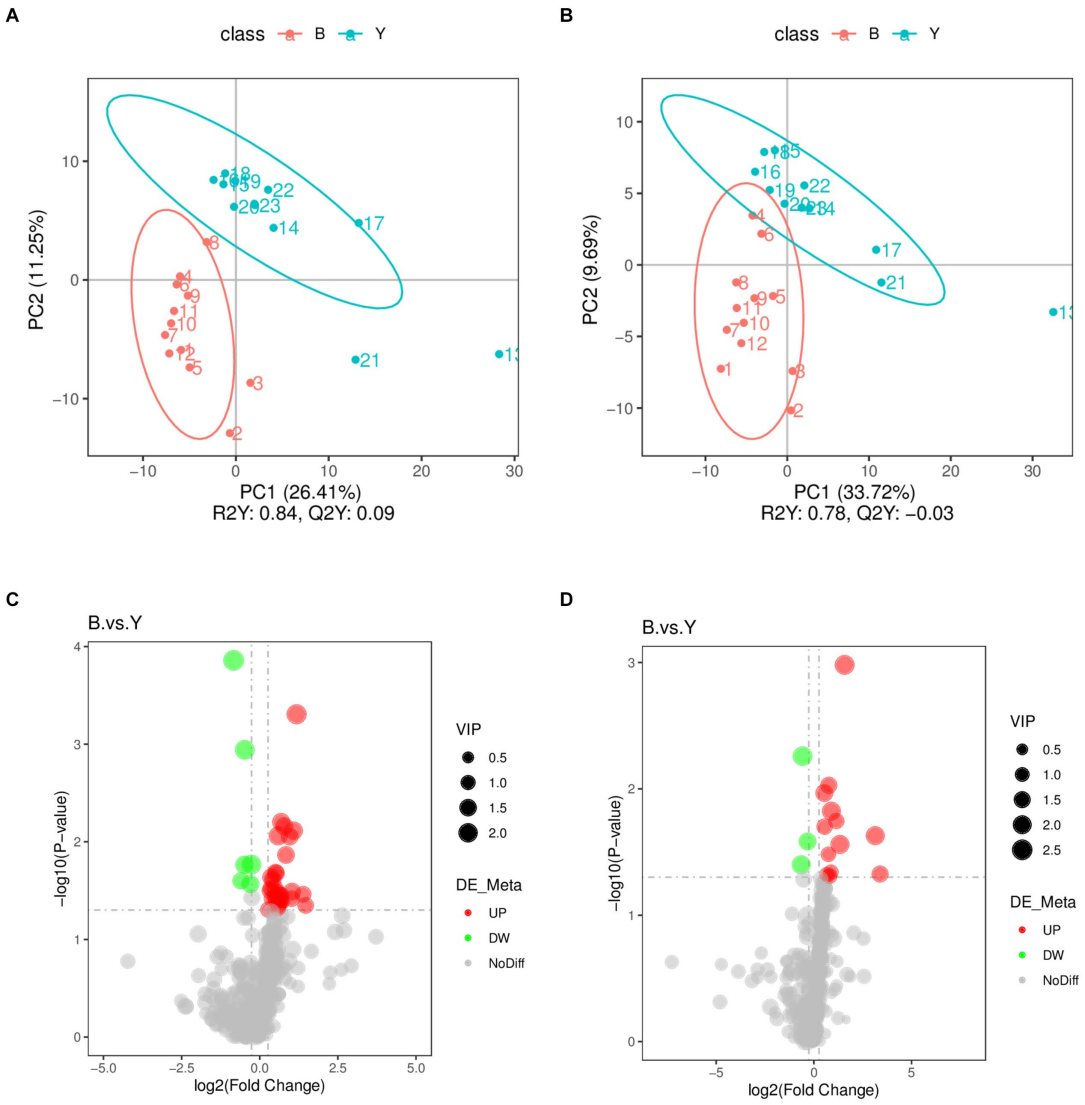
Screening of differential metabolites in Huyang ewe plasma samples. PLS-DA scatter plot of the experimental and control groups in positive ion modes **(A)** and negative ion modes **(B)**. The abscissa is the sample’s score on the first principal component, and the ordinate is the sample’s score on the second principal component. R2Y represents the interpretation rate of the model, Q2Y is used to evaluate the predictive ability of the PLS-DA model, and when R2Y is greater than Q2Y, the model is well established. Volcano plot of differential metabolites in positive ion mode **(C)** and the negative ion mode **(D)**. The gray plot shows there is no difference. The red plots show upregulated endogenous metabolites, while the green plots show downregulated endogenous metabolites. VIP value represents the important projection value of the metabolite obtained in the PLS-DA model compared to this group. B: experimental group; Y: control group.

Partial Least Squares Discriminant Analysis established a relationship model between metabolite expression and sample category, facilitating sample category prediction. To discriminate the differential metabolites between the two groups, supervised PLS-DA was performed to find different metabolites. As shown in Figure 2, the prediction parameters of the PLS-DA evaluation model exhibit R2Y values of 0.84 and 0.78 in positive and negative ion modes (Figures 2A,B), with the Q2Y values of 0.09 and − 0.03, respectively. The higher R2Y values compared to Q2Y in both modes indicate the reliability of the model used in this study.

**Figure 3:**
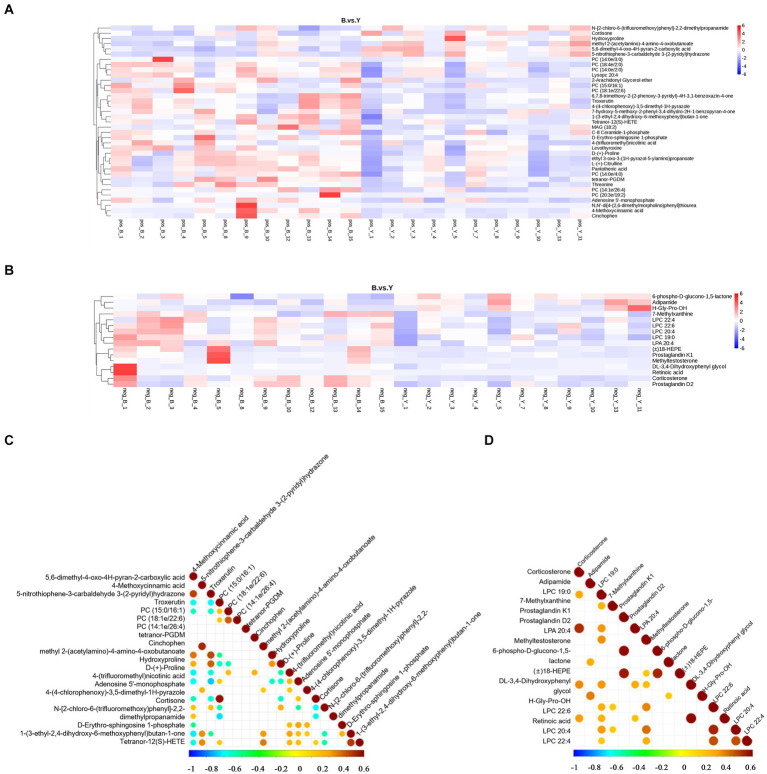
Analysis of differential metabolites in Huyang ewe plasma samples. Heat map of differential metabolite levels in positive ion mode **(A)** and the negative ion mode **(B)**. The vertical direction was the clustering of metabolites. The correlation diagram of differential metabolites in the positive ion model **(C)** and negative ion mode **(D)**. Pearson’s correlation coefficient between all metabolites was calculated to analyze the correlation between each metabolite. When the linear relationship between the two metabolites is strengthened, the positive correlation tends to be 1, and the negative correlation tends to be −1. At the same time, the statistical test of significance was carried out on the correlation analysis of metabolites, and the threshold of significant correlation was selected as the significance level *p* value <0.05.

Metabolomic data were analyzed using FC, *p* value, and VIP in univariate analysis. Differential metabolites were subsequently identified based on VIP > 1.0, FC > 1.2 or FC < 0.833, and *p* < 0.05. A total of 53 metabolites exhibiting significant differences were identified: 37 in positive ion mode, comprising 31 upregulated and six downregulated metabolites, and 16 in negative ion mode, with 13 upregulated and three downregulated (Table 2; Figures 2C,D). As shown in Figures 3A,B, the heat map showed all differential metabolites in positive and negative ion modes. The top 10 differential metabolites in positive ion mode are N,N′-di[4-(2,6-dimethylmorpholino)phenyl]thiourea, phosphatidylcholine (20:3e/19:2), 4-methoxycinnamic acid, phosphatidylcholine (18:1e/22:6), 2-arachidonyl glycerol ether, phosphatidylcholine (14:0e/3:0), tetranor-pgdm, cinchophen, phosphatidylcholine (15:0/16:1), and monoacylglycerol (18:2). The top 10 differential metabolites in negative ion mode are retinoic acid, methyltestosterone, corticosterone, (±) 18-hydroxyeicosa-5,8,11,14,16-pentaenoic acid (18-HEPE), prostaglandin D2, prostaglandin K1, lysophosphatidylcholine (LPC) 22:6, LPC 22:4, LPC 19:0, and DL-3,4-dihydroxyphenyl glycol. Among them, the metabolic levels of cortisone and hydroxyproline significantly decreased, and the pantothenic acid level significantly increased in the experimental group in positive ion mode. In negative ion mode, the metabolic levels of corticosterone and prostaglandin D2 of the experimental group significantly increased (Figure 3B). Correlation analysis of differential metabolites was performed using Pearson correlation analysis (Figures 3C,D). In positive ion mode, the metabolic levels of hydroxyproline had a significant positive correlation with cortisone and 5-nitrothiophene-3-carbaldehyde 3-(2-pyridyl) hydrazone (Figure 3C, *p* < 0.05). The metabolic levels of corticosterone and prostaglandin D2 were significantly positively correlated in negative ion mode (Figure 3D, *p* < 0.05).

**Table 2:** Differential metabolites detected in Huyang ewe plasma samples under positive and negative ion modes.

Compared samples	No. Total Ident	No. Total Sig	No. Sig Up	No. Sig Down
B vs. Y_pos	464	37	31	6
B vs. Y_neg	376	16	13	3

B, Experimental group; Y, Control group. Compared samples: Compare the pairs of samples, for the former than the latter; No. Total Ident, Total metabolite identification results; No. Total Sig, The total number of metabolites with significant differences; No. Sig Up, The total number of significantly upregulated metabolites; and No. Sig Down, The total number of significantly downregulated metabolites.

### 3.4 The impact of GnRH on metabolic pathways

**Figure 4:**
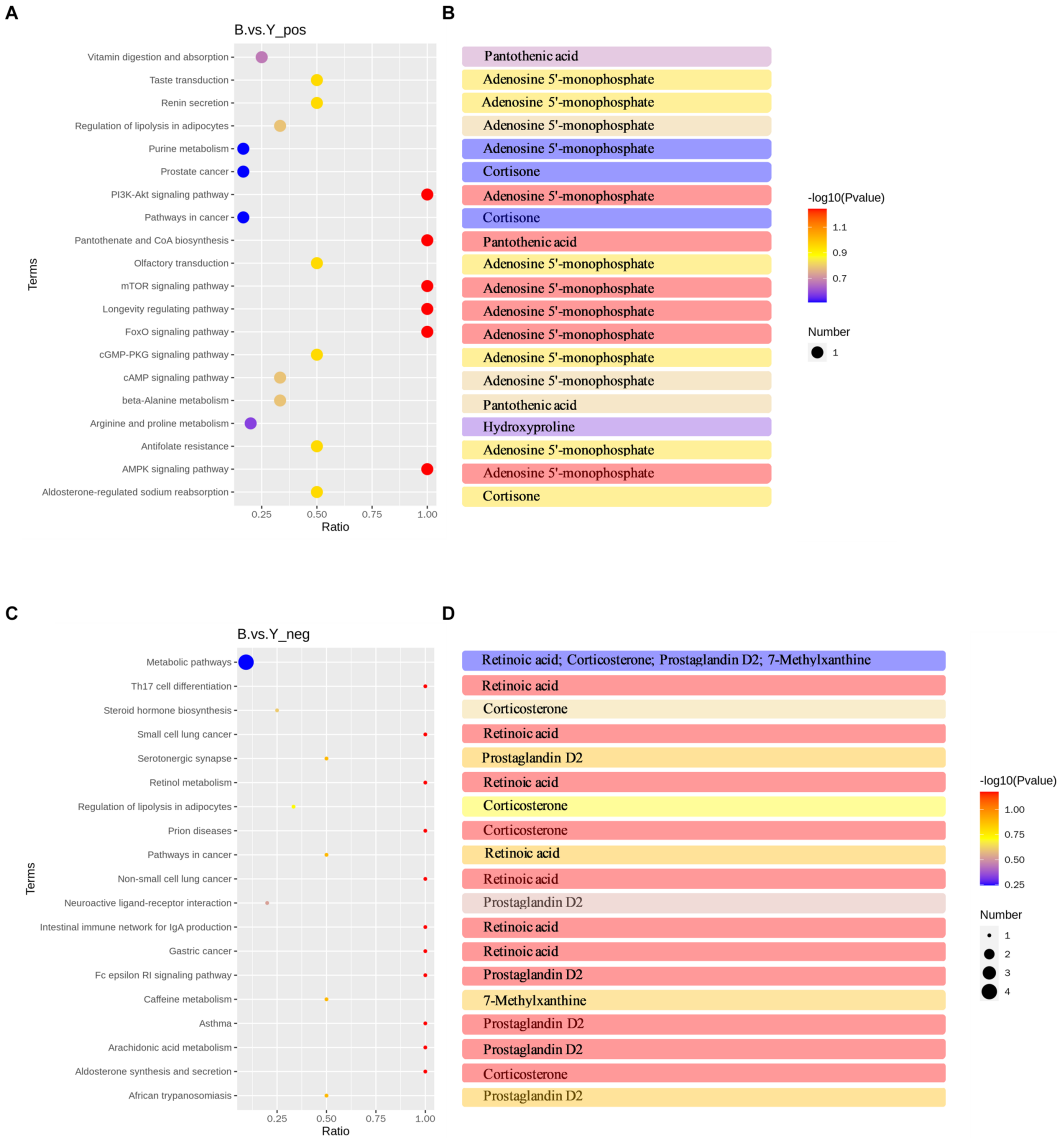
KEGG pathway analysis of differential metabolites in Huyang ewe plasma samples. **(A)** Top 20 KEGG enrichment pathways of differential metabolites in positive ion mode. **(B)** Metabolites have significant differences in each pathway in the positive ion mode. **(C)** Top 19 KEGG enrichment pathways of differential metabolites in the negative ion mode. **(D)** Metabolites have significant differences in each pathway in the negative ion mode. The horizontal coordinate is *x*/*y* (the number of differential metabolites in the corresponding metabolic pathway/the number of total metabolites identified in the pathway). The higher the value, the higher the enrichment degree of differential metabolites in the pathway. The color of the dot represents the *p* value of the hypergeometric test, and the smaller the value, the greater the reliability and statistical significance of the test. The size of the dot represents the number of differential metabolites in the corresponding pathway, and the larger the dot, the more differential metabolites in the pathway.

To explore the physiological functions of metabolites affected by GnRH treatment, KEGG pathway enrichment analysis was performed on 53 differential metabolites in positive and negative ion modes. KEGG pathway enrichment analysis revealed 22 significantly enriched pathways in positive and 19 in negative ion modes. These pathways belonged to five modules: metabolism, genetic information processing, cellular processes, environmental information processing, and organismal systems. The enriched pathways of differential metabolites in positive ion mode mainly included pantothenate and CoA biosynthesis, Foxo signaling pathway, mTOR signaling pathway, PI3K-Akt signaling pathway, cGMP-PKG signaling pathway, aldosterone-regulated sodium reabsorption, and cAMP signaling pathway (Figure 4A). Significantly downregulated hydroxyproline was enriched in the arginine and proline metabolism pathway (Figure 4B).

**Figure 5:**
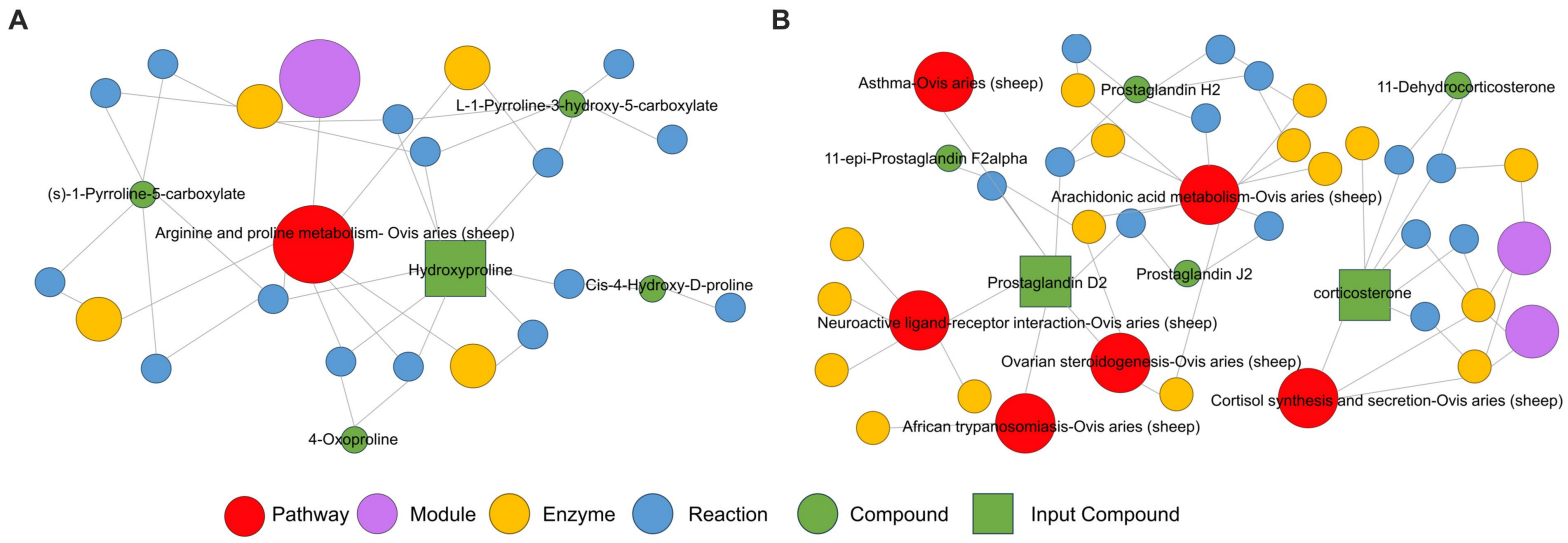
KEGG enrichment pathway network diagram of hydroxyproline, corticosterone, and prostaglandin D2 metabolites in the positive ion mode **(A)** and negative ion mode **(B)** of Huyang ewe plasma samples. In this network plot, the red dot represents a metabolic pathway, the yellow dot represents substance-related regulatory enzyme information, the green dot represents a background substance of a metabolic pathway, the purple dot represents a class of substance molecular module information, the blue dot represents a substance chemical interaction reaction, and the green square represents the difference substance obtained from this comparison.

Conversely, in negative ion mode, differential metabolites were primarily enriched in aldosterone synthesis and secretion, arachidonic acid metabolism, neuroactive ligand-receptor interaction, steroid hormone biosynthesis, and Th17 cell differentiation, among other metabolic pathways (Figure 4C). Significantly upregulated corticosterone was primarily enriched in the aldosterone synthesis and secretion pathway and the steroid hormone biosynthesis pathway (Figure 4D). Additionally, there is a significant increase in the metabolic content of prostaglandin D2 in the arachidonic acid metabolism pathway and the interaction of neuroactive ligands with receptors (Figure 4D). Moreover, a KEGG regulatory network was constructed utilizing the differential metabolites (Figures 5A,B). The figure visually represents changes in metabolites such as hydroxyproline, corticosterone, and prostaglandin D2. It also displays the intersections and biological processes related to these metabolites and their corresponding pathways.

## 4 Discussion

Synchronization and artificial insemination techniques have been widely used in sheep reproduction, and GnRH treatment during insemination has become a relatively common practice in the sheep industry (26). However, the impact of GnRH on embryo development and implantation remains debated among various studies. Our study compared the 40-day pregnancy rates between GnRH-treated and control sheep. The results indicated that the pregnancy rate in the experimental group (72.5%, *n* = 69) was significantly lower than that in the control group (82.9%, *n* = 41) (*p* < 0.05).

The pregnancy results obtained in our control group are acceptable, which is consistent with the results of conventional progesterone + eCG synchronization treatment and artificial insemination (27). Previous studies have shown that treatment with GnRH 24–48 h after progesterone withdrawal in sheep does not enhance the pregnancy rate (11, 14). Corriedale sheep were treated with two prostaglandins to induce synchronization and FTAI with GnRH 24 h after the second prostaglandin used decreased the pregnancy rate following artificial insemination. However, treatment with GnRH 36 h later had no significant effect on the pregnancy rate (14). Additionally, administering GnRH simultaneously with single-timed insemination can potentially decrease the pregnancy rate (28). Similarly, GnRH treatment 36 h after progesterone withdrawal and performing insemination 48 h later did not lead to a significant improvement in the pregnancy rate in goats (6). In the MOET process, the receptors treated with GnRH analog Folligon also achieved a low pregnancy rate of 43.2% (29). These previous study results are consistent with the results in this study.

Moreover, metabolomics analysis was performed to investigate the potential reasons behind the lower pregnancy rate observed in GnRH-treated sheep. Metabolomics employs comprehensive analysis through positive and negative ion modes, aiming to capture the diverse metabolites within the organism. The positive ion mode tends to detect alkaline metabolites, while the negative ion mode focuses on acidic substances (30). This study conducted metabolite screening and discussion separately in two modes. Significantly, differential metabolites were screened, including hydroxyproline in positive ion mode and corticosterone and prostaglandin D2 in negative ion mode. Hydroxyproline, a distinctive amino acid found in collagen, is a marker for collagen degradation (31, 32). It comprises approximately 13% of collagen’s total amino acid content, making it crucial for maintaining collagen’s structure and function. During pregnancy, the uterine collagen content progressively rises, leading to a corresponding increase in hydroxyproline levels (33). Currently, the uterus and genital tract’s extracellular matrix contain abundant collagen, essential for supporting the growing fetal load and maintaining gestation (34). Metabonomics results revealed that hydroxyproline metabolite was significantly downregulated in the arginine and proline metabolism pathway (*p* < 0.05) in Huyang ewes treated with GnRH during the peri-implantation period. This downregulation may decrease collagen synthesis, affecting endometrial tissue’s stability and mechanical strength and potentially causing uterine tissue damage or dysfunction. Such conditions are unfavorable for establishing a suitable environment for embryo implantation. Furthermore, studies have demonstrated that collagen rapidly degrades after cows give birth, decreasing uterine size and weight as part of the uterine involution process (35). In conclusion, a significant reduction in hydroxyproline level during the peri-implantation period is detrimental to embryo implantation in the uterus, ultimately decreasing the pregnancy rate in Huyang ewes.

The results of this study indicate that the metabolism of corticosterone in the aldosterone synthesis and secretion pathway significantly increased (*p* = 0.01) in Huyang ewes treated with GnRH. Corticosterone, produced primarily by the adrenal cortex, plays a crucial role in the stress response (36). Studies have shown that up-regulated corticosterone levels can inhibit embryonic development and decrease the number of inner cell mass cells, compromising embryo quality and affecting pregnancy rates (37). Zheng et al. continuously use exogenous corticosterone in mice during pregnancy days 1–4. Compared to the control group, using corticosterone resulted in a sharp decrease in phosphorylated Stat3, affecting endometrial receptivity and significantly reducing the number of implantation sites on day 5 (38). Similarly, studies on a mouse model of corticosterone excess have shown that elevated corticosterone levels affect female fertility by influencing the uterus rather than the oocytes (39). These findings are consistent with our results. So, we thought that treatment with GnRH during insemination significantly increases corticosterone levels in Huyang ewes before implantation, thereby affecting the uterine environment before implantation, disrupting endometrial receptivity, and ultimately decreasing the pregnancy rate.

Prostaglandin D2 (PGD2) belongs to the prostaglandin family and is synthesized from arachidonic acid (AA) via the action of cyclooxygenase (COX) (40). Metabolomic analyses indicated a significant increase in prostaglandin D2 within the arachidonic acid metabolism pathway (*p* = 0.017). Although PGD2 promotes luteal regression, its effect is less pronounced than that of Prostaglandin F2 alpha (PGF2α). Nonetheless, PGF2α and PGD2 exhibit synergistic effects in promoting luteal regression by effectively expanding blood vessels and increasing inflow to luteal tissue, which facilitates the influx of additional PGF2α synthesized in the endometrium and accelerates the luteal regression process (41). In addition, PGs serve as key mediators in the study of inflammation (42). Under inflammatory conditions or other stimuli, the expression of COX-2 is significantly upregulated, further promoting the synthesis of PGs to a notable degree (43). The induction of intrauterine inflammation can negatively impact the implantation and development of embryos within the uterus (44). Studies have revealed the presence of PGD2 in human endometrial and uterine smooth muscle tissues, where it stands out as the most potent substance within the PG family for augmenting blood flow to the endometrium and uterine muscles (45). The significant elevation of PGD2 can trigger and expedite uterine smooth muscle contraction frequency, ultimately decreasing embryo survival rate (46). Consequently, the imbalance of PGD2 levels in ewes adversely affects embryo implantation, resulting in a decreased pregnancy rate in the experimental group.

## 5 Conclusion

In summary, administering a single dose of the GnRH agonist triptorelin 48 h after sponge removal reduces the pregnancy rate in Huyang ewes after insemination. Metabolomic analysis revealed a significant decrease in hydroxyproline levels and a significant increase in corticosterone and prostaglandin D2 levels, which are linked to a decreased pregnancy rate in Huyang ewes.

## Data availability statement

The data presented in the study are deposited in the MetaboLights repository, accession number MTBLS10318.

## Ethics statement

The animal study was approved by Animal Care and Use Committee of the Hebei Agricultural University. The study was conducted in accordance with the local legislation and institutional requirements.

## Author contributions

JZ: Data curation, Formal analysis, Investigation, Methodology, Project administration, Software, Validation, Writing – original draft, Writing – review & editing. SS: Data curation, Formal analysis, Investigation, Methodology, Resources, Writing – original draft. XB: Data curation, Formal analysis, Investigation, Methodology, Visualization, Writing – original draft. NY: Data curation, Methodology, Resources, Writing – review & editing. YL: Data curation, Methodology, Resources, Writing – review & editing. XW: Conceptualization, Formal analysis, Funding acquisition, Methodology, Supervision, Validation, Visualization, Writing – original draft, Writing – review & editing. XL: Conceptualization, Data curation, Funding acquisition, Methodology, Resources, Supervision, Validation, Visualization, Writing – original draft, Writing – review & editing.
